# Pancreatic volume and immune biomarkers predict checkpoint inhibitor–associated autoimmune diabetes in humans

**DOI:** 10.1172/JCI192938

**Published:** 2025-11-20

**Authors:** Linda Wu, John M. Wentworth, Christopher Liddle, Nicole Fewings, Matteo Carlino, David A. Brown, Roderick Clifton-Bligh, Georgina V. Long, Richard A. Scolyer, Nicholas Norris, Sarah C. Sasson, Venessa H.M. Tsang, Alexander M. Menzies, Jenny E. Gunton

**Affiliations:** 1 Centre for Diabetes, Obesity and Endocrinology Research, Westmead Institute for Medical Research, Westmead, NSW, Sydney, Australia.; 2 Department of Diabetes and Endocrinology, Westmead Hospital, NSW, Sydney, Australia.; 3 Department of Diabetes and Endocrinology, Royal North Shore Hospital, NSW, Sydney, Australia.; 4 Westmead Institute for Medical Research, University of Sydney, NSW, Sydney, Australia.; 5 Department of Diabetes and Endocrinology, Royal Melbourne Hospital and Walter and Eliza Hall Institute of Medical Research, Melbourne, Australia.; 6 Melanoma Institute Australia, NSW, Sydney, Australia.; 7 NSW Health Pathology, Westmead, NSW, Sydney, Australia.

**Keywords:** Autoimmunity, Endocrinology, Oncology, Cancer immunotherapy, Diabetes, Melanoma

## Abstract

**BACKGROUND:**

Checkpoint inhibitor–associated autoimmune diabetes mellitus (CIADM) is a rare but life-altering complication of immune checkpoint inhibitor (ICI) therapy. Biomarkers that predict type 1 diabetes (T1D) are unreliable for CIADM.

**AIM:**

In the present study, we sought to identify biomarkers for the prediction of CIADM.

**METHODS:**

From our prospective biobank, we identified 14 patients with CIADM who had metastatic melanoma treated with anti–programed antibody death 1 (anti–PD-1) with or without anti–cytotoxic T lymphocyte–associated antibody protein 4 (anti-CTLA4). Controls were selected from the same biobank, matched 2:1. Pretreatment, on-ICI, and post-CIADM serum and PBMCs were analyzed. Serum was analyzed for T1D autoantibodies, C-peptide, glucose, and cytokines. PBMCs were profiled using flow cytometry. Pancreatic volume was measured using CT volumetry.

**RESULTS:**

Before treatment, patients with CIADM had smaller pancreatic volume (27% reduction, *P* = 0.044) and higher anti–glutamic acid decarboxylase autoantibody (anti-GAD) titers (median 2.9 vs. 0, *P* = 0.01). They had significantly higher baseline proportions of Th17 cells (*P* = 0.03), higher CD4^+^ central memory cells (*P* = 0.04), and lower naive CD4^+^ T cells (*P* = 0.01). With ICI treatment, greater declines in pancreatic volume were seen in patients with CIADM (*P* < 0.0001). Activated CD4^+^ T cell subsets increased significantly in CIADM and controls with immune-related adverse effects (IRAEs) but not in controls without IRAEs. Using only pretreatment results, we found that pancreatic volume, anti-GAD antibody titers, and the baseline immune flow profile were highly predictive of CIADM development, with an AUC of greater than 0.96.

**CONCLUSIONS:**

People who develop CIADM are immunologically predisposed and have antecedent pancreatic and immunological changes that accurately predict disease with excellent sensitivity. These biomarkers could be used to guide ICI use, particularly when planning treatment for low-risk tumors.

**FUNDING:**

National Health and Medical Research Council (NHMRC) Investigator grants 2033228, 2009476, and 2007839.

## Abstract 2

We have identified excellent predictors for risk of developing checkpoint-inhibitor autoimmune diabetes mellitus. These results may be used to reduce risk of CIADM in people receiving cancer therapy.

## Graphical abstract

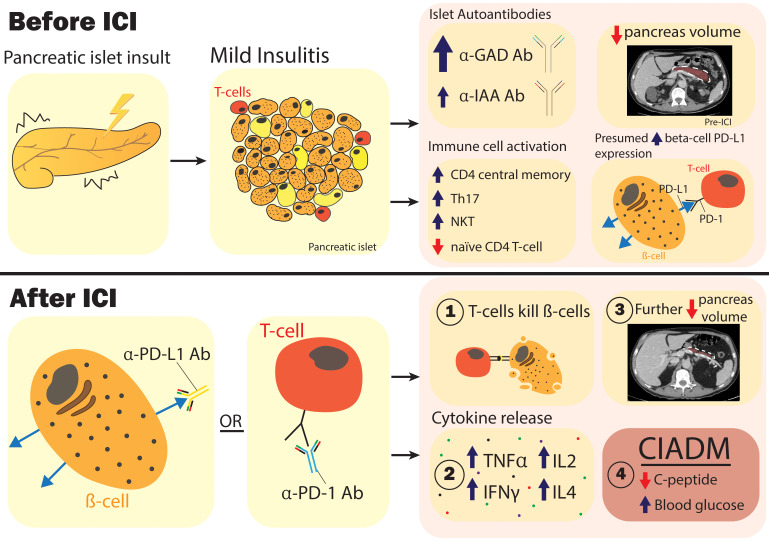

## Introduction

Immune checkpoint inhibitors (ICIs) have transformed the treatment for many malignancies since their initial introduction in the treatment of melanoma. Eleven ICIs are now FDA approved for at least 43 indications in a wide range of malignancies (1). While primarily used in the setting of metastatic cancer, recent studies also demonstrate benefits in the adjuvant and neoadjuvant settings (2, 3).

As the use of ICIs increases, the corresponding incidence of immune-related adverse effects (IRAEs) will also rise. Among these, checkpoint inhibitor–related autoimmune diabetes mellitus (CIADM; also termed ICI-DM) is of particular interest, given the major, lifelong physical and psychosocial effects of insulin-requiring diabetes and the propensity for fulminant onset with a high risk of diabetic ketoacidosis. We have previously demonstrated that CIADM bears similarities to its de novo counterpart, type 1 diabetes (T1D), with respect to insulin deficiency and lifelong insulin dependency. However, there are also distinct differences, including a high prevalence of T1D antibody negativity and fulminant β cell failure, thus warranting separate diagnostic criteria and evaluation (4). CIADM develops in 0.4%–1.9% of people treated with therapies directed against programed death 1 (PD-1) or programed death ligand 1 (PD-L1) (5–9).

The ability to estimate an individual’s risk of developing serious IRAEs prior to starting ICI would inform treatment decisions, especially in the adjuvant setting and where effective alternative treatments are available. Studies show that the overall risk of any IRAEs is associated with higher baseline CD4^+^ counts (10), early Treg expansion (11), increased CD8^+^ clonal responses (12), a more diverse T cell repertoire (13), higher cytokine levels at baseline and early in treatment (11, 14, 15), neutrophil/lymphocyte ratio (16), and genetic variants (17).

In T1D, anti-islet autoantibodies predict the risk of disease with high accuracy (18). HLA haplotypes are also strongly linked to T1D risk, with approximately 95% of people having high-risk HLA DR3 and/or DR4, and genetic risk scores are available to further delineate the risk (19). A decline in pancreatic volume is associated with a risk of progression from preclinical to overt T1D (20). Flow cytometry shows differences in CD4^+^ T follicular helper cells, Tregs, and naive and Th17 cell subsets associated with T1D onset (21–25). Islet-specific autoreactive T cells are a promising T1D biomarker, but assays are subject to HLA-type restrictions (26, 27).

The aim of this study was to identify potential biomarkers for CIADM risk prior to commencement of ICI therapy and early during treatment. A secondary aim was to identify biomarkers for risk prediction after ICI commencement but before CIADM onset. We compared control participants with CIADM patients before ICI treatment, early during treatment, and after CIADM diagnosis.

## Results

**Figure 1:**
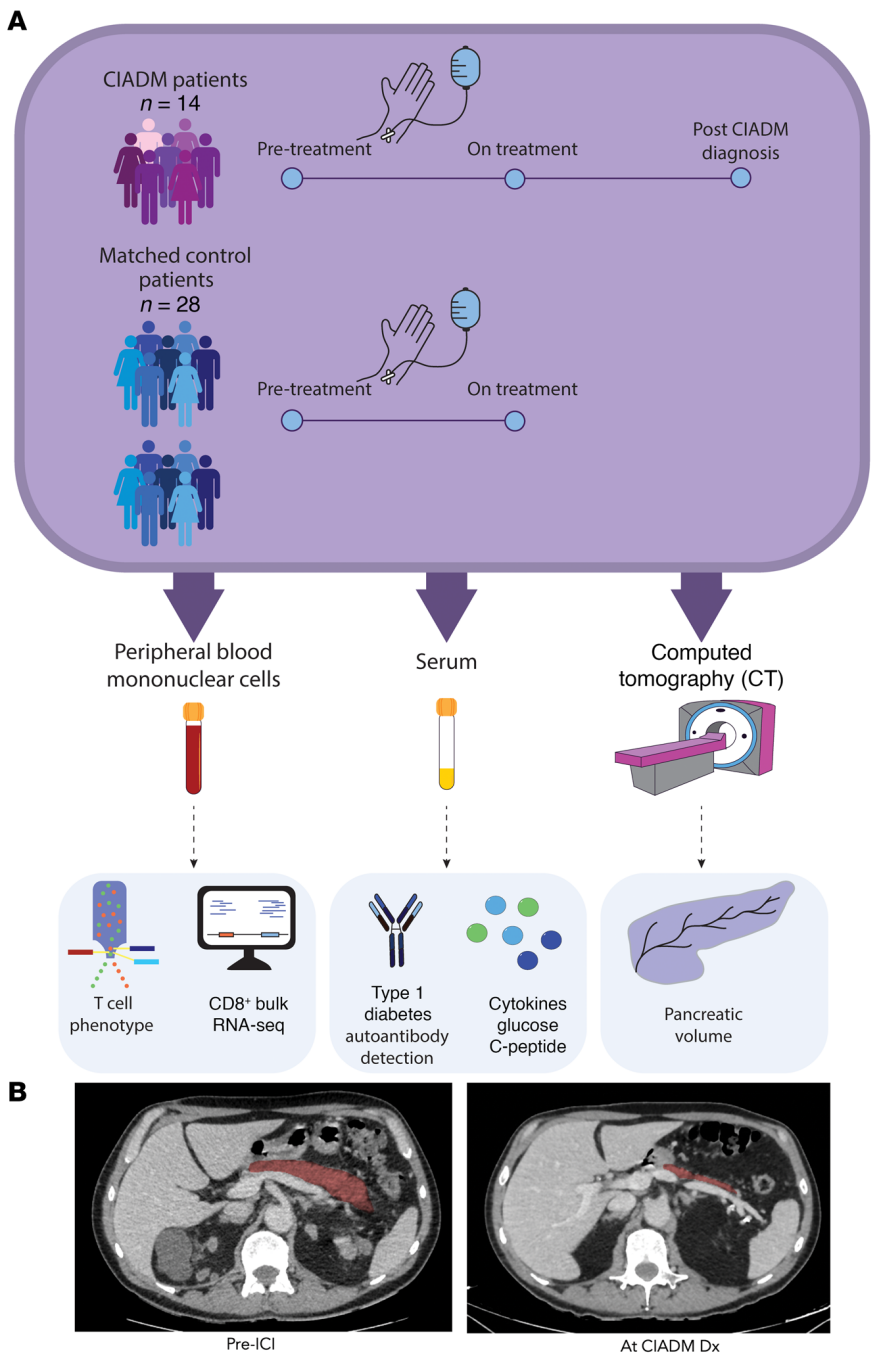
(A) Summary of methodology. (**B**) Representative CT scans of a patient with CIADM prior to ICI therapy and at the time of CIADM diagnosis (red indicates the pancreatic area).

A summary of the methods is shown in Figure 1, and the flow cytometry gating strategy is shown in Supplemental Figure 1; supplemental material available online with this article; https://doi.org/10.1172/JCI192938DS1 Fourteen patients with CIADM and 28 matched controls — all treated with ICI — were included. All patients had metastatic melanoma. Of the total number of samples sought, 2 PBMC samples were not available for patients with CIADM at the pretreatment time point. Baseline characteristics are shown in Table 1. Prior exposure to other anticancer treatments was predominantly dabrafenib and trametinib therapy.

### Subclinical anti-GAD and anti-IAA antibody levels are associated with CIADM.

**Figure 2:**
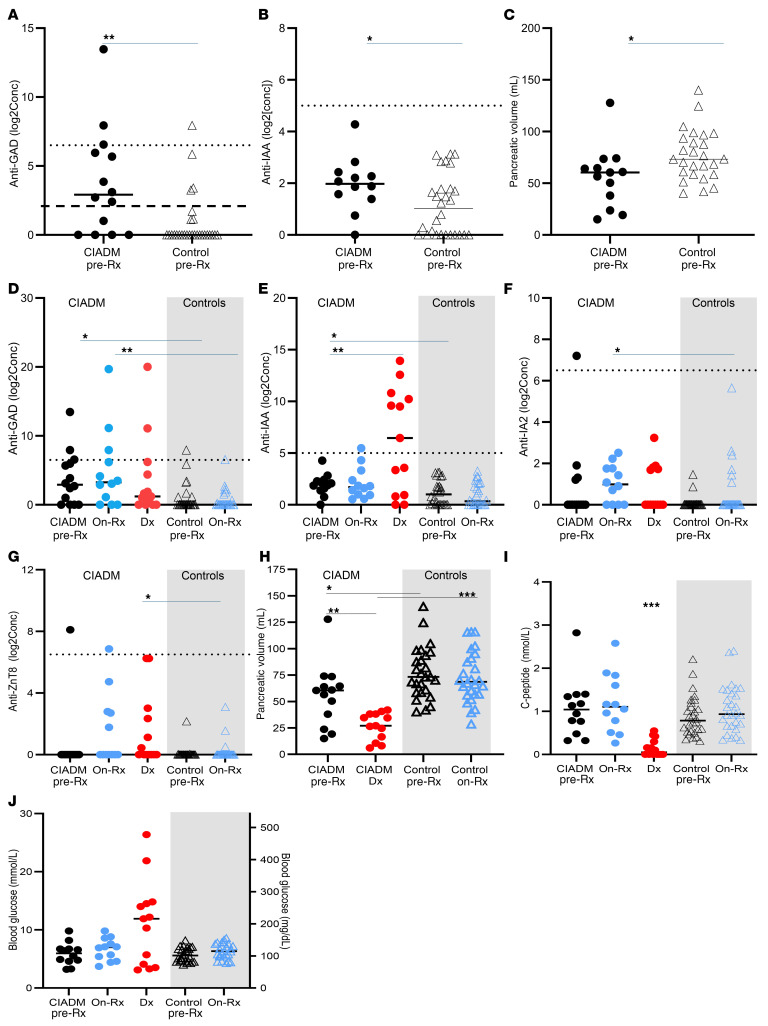
Antibody levels, pancreatic volume, C-peptide levels, and glucose levels. (**A**) Anti-GAD antibody levels before ICI treatment (pre-Rx) in controls and patients with CIADM. (**B**) Pre-ICI anti-IAA levels. (**C**) Pre-ICI pancreatic volume. (**D**) Anti-GAD antibody levels before and on ICI. (**E**) Anti-IAA antibody levels before and on ICI. (**F**) Anti-IA2 antibody levels before and on ICI. (**G**) Anti-ZnT8 antibody levels before and on ICI. (**H**) Pancreatic volume before and on ICI. (**I**) C-peptide levels before and on ICI. (**J**) Blood glucose levels before and on ICI. Lines indicate median. Dotted lines at **A**, **B**, and **D**–**G** indicate thresholds for positive. *P < 0.05, **P < 0.01, and ***P < 0.0001 for the indicated comparisons, by Mann-Whitney *U* test (**A**–**C**) and Kruskal-Wallis corrected for multiple comparisons with Dunn’s test (**D**–**J**). conc, concentration; Dx, diagnosis.

Before ICI treatment, glutamic acid decarboxylase autoantibody (anti-GAD) titers were significantly higher in patients with CIADM than in controls (Figure 2A, *P* = 0.002, Mann-Whitney *U*). Despite the higher antibody levels with this sensitive assay, only 2 patients (14%) had levels above the reference range for anti-GAD before ICI exposure, with 1 patient showing levels at, but not above, the top of the reference range (dotted line) and an additional 6 patients having levels above the threshold of detection (dashed line), giving a total of 64% of patients with CIADM having detectable levels compared with 4 ICI-treated controls who did not develop diabetes (14%, *P* < 0.001 vs. patients with CIADM, χ^2^ with Yates correction).

Figure 2B shows that anti-insulin autoantibody (IAA) titers were also significantly higher in patients with CIADM before treatment than in pretreatment control individuals (*P* = 0.048, Mann-Whitney *U*). Seven patients with CIADM were above the threshold of detection for the assay (54%) compared with 6 of 28 controls (21%, *P* = 0.038). As insulin exposure is known to provoke IAA development, it should be noted that no patients had exposure to insulin prior to ICI treatment. However, the rise of IAA seen in patients with CIADM after diagnosis and insulin treatment was consistent with this also being common after T1D diagnosis.

Autoantibody positivity above the clinical test threshold was not significantly associated with an increased risk of presentation with diabetic ketoacidosis or earlier with CIADM diagnosis.

### Pancreatic volume is lower in patients with CIADM before ICI treatment.

Pancreatic volume was measured using CT scans. Pancreatic volume on CT scans was lower before ICI exposure in individuals who went on to develop CIADM than in controls (median 60 vs. 73 mL, Figure 2C, *P* = 0.019). All but 1 patient who went on to develop CIADM had a baseline pancreatic volume of less than 75 mL (92%) compared with 14 of 28 controls (50%, *P* = 0.0007 by χ^2^ with Yates correction).

### Antibody levels and pancreatic volume change with ICI treatment.

After ICI exposure, anti-GAD titers were significantly higher in people who went on develop CIADM than in on-treatment controls (*P* = 0.008, Kruskal-Wallis with Dunn’s correction for multiple comparisons, Figure 2D). Anti-IAA titers tended to be higher on-ICI in those who developed CIADM than in controls on ICI, but this did not remain significant after correction for multiple comparisons (*P* = 0.09, Figure 2E).

Figure 2F shows that anti-insulinoma antigen 2 (IA2) titers were higher in CIADM patients on ICI than in controls on ICI (*P* = 0.045). In Figure 2G, anti–zinc transporter 8 (ZnT8) titers were higher in patients at CIADM diagnosis than in on-ICI controls (*P* = 0.04) but did not differ before diabetes onset.

Pancreatic volume was profoundly reduced at CIADM diagnosis compared with before ICI treatment (*P* < 0.01) and was substantially lower than in on-ICI controls (*P* < 0.0001, Figure 2H). Most patients with CIADM did not undergo an on-treatment scan prior to the onset of CIADM.

### Pretreatment and prediabetes glucose and C-peptide levels do not predict future CIADM development.

Insulin secretion was assessed by measuring C-peptide and concurrent glucose levels. People who went on to develop CIADM did not have lower C-peptide levels before ICI treatment or when on ICIs before CIADM diagnosis (Figure 2I).

C-peptide levels fell from a median of 1.0 (IQR 0.6–1.4) nmol/L pre-ICI and 1.1 (0.6–1.8) on ICI to 0.05 (0–0.3) nmol/L after diagnosis for patients with CIADM. In control participants, C-peptide levels remained normal (Figure 2I).

Formal blood glucose levels were not available for all patients with CIADM after diagnosis, before commencement of insulin, and the available glucose levels did not differ significantly (Figure 2J). Overall, neither C-peptide nor serum glucose levels are predictive of future CIADM.

**Figure 3:**
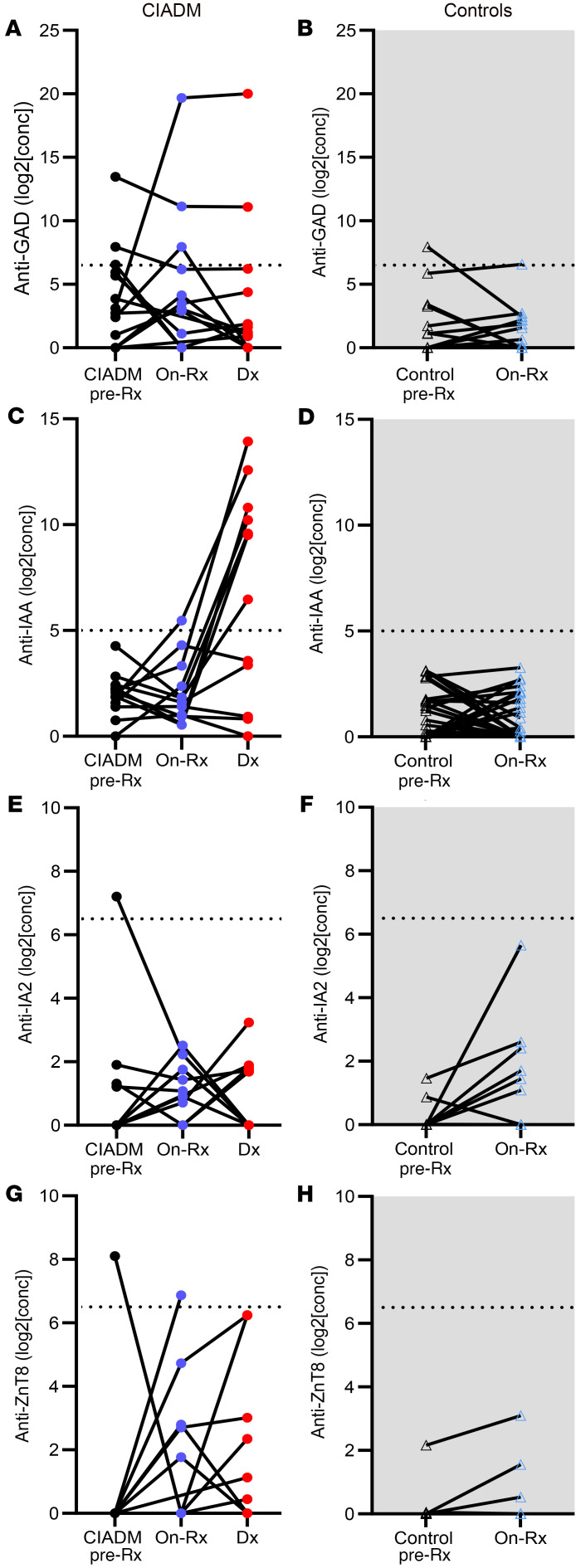
Changes in antibody titers over time. (**A** and **B**) Anti-GAD antibody levels. (**C** and **D**) Anti-IAA antibody levels. (**E** and **F**) Anti-IA2 antibody levels. (**G** and **H**) Anti-ZnT8 antibody levels. Note the majority of people who did not develop CIADM (controls) were 0 to 0 titers for all antibodies except IA2, so their results align across the *x* axis. We note that developing insulin autoantibodies after commencing insulin therapy is common, as was observed in the CIADM group after starting insulin.

Changes in antibody titers with ICI treatment among individuals were examined to assess whether this may be an independent predictor of CIADM (Figure 3). No pattern of antibody change during treatment significantly predicted CIADM.

### Altered circulating cytokine levels are associated with CIADM.

**Figure 4:**
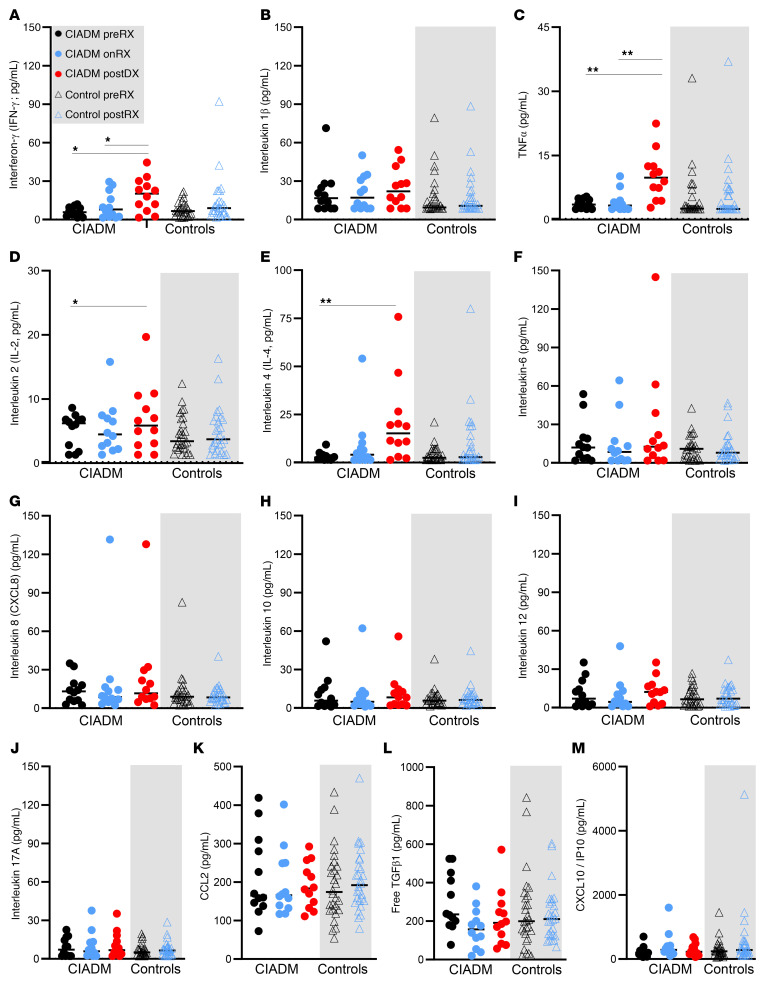
Circulating cytokine levels. (**A**) IFN-γ, (**B**) IL-1β, (**C**) TNF-α, (**D**) IL-2, (**E**) IL-4, (**F**) IL-6, (**G**) IL-8, (**H**) IL-10, (**I**) IL-12, (**J**) IL-17A, (**K**) CCL2, (**L**) free TGF-β1, and (**M**) CXCL10 levels. *P < 0.05 and **P < 0.01 for the indicated comparisons, by Kruskal-Wallis test with Dunn’s correction for multiple comparisons. Lines indicate the median.

Figure 4 depicts circulating cytokine concentrations at the different time points in patients with CIADM and controls. No cytokines showed differential expression before ICI therapy.

IFN-γ, IL-1β, and TNF-α are the cytokines most classically associated with T1D. IFN-γ levels were elevated at CIADM diagnosis compared with before ICI or on ICI (Figure 4A, *P* < 0.05). There were no significant differences in IL-1β levels (Figure 4B). Figure 4C shows that TNF-α levels also rose significantly at the time of CIADM diagnosis compared with baseline or on-ICI levels in patients with CIADM.

IL-2 and IL-4 levels were also both significantly higher at CIADM diagnosis than at baseline (Figure 4, D and E, *P* < 0.05). IL-6, IL-8 (also called CXCL8), IL-10, IL-12, and IL-17A levels did not differ between groups (Figure 4, E–J), nor did CCL2 (chemokine C-C motif ligand 2), free TGF-β, or C-X-C motif chemokine ligand 10 (CXCL10) levels (Figure 4, K–M).

### Cytokine levels predict IRAEs.

**Figure 5:**
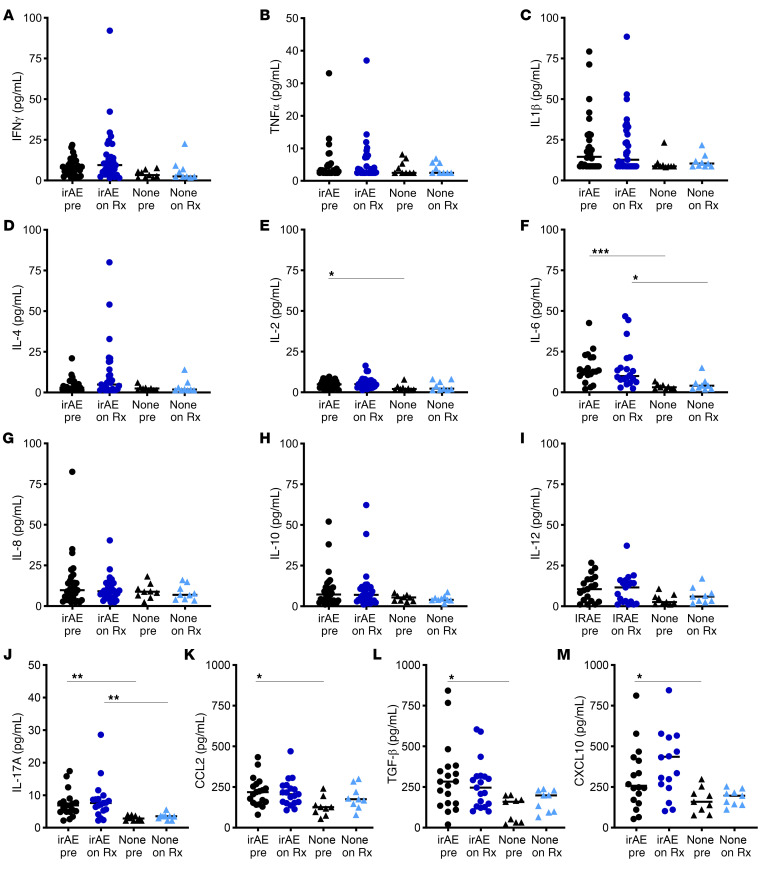
Cytokine levels in patients without IRAEs before and on ICI and in people with IRAEs before and after ICI. (**A**) IFN-γ, (**B**) IL1-β, (**C**) TNF-α, (**D**) IL-2, (**E**) IL-4, (**F**) IL-6, (**G**) IL-8, (**H**) IL-10, (**I**) IL-12, (**J**) IL-17A, (**K**) CCL2, (**L**) free TGF-β1, and (**M**) CXCL10 levels. *P < 0.05, **P < 0.01, and ***P < 0.001 for the indicated comparisons by Kruskal-Wallis testing with Dunn’s correction for multiple comparisons. Lines indicate the median.

The 9 control participants who did not develop any known IRAE were compared with people who developed CIADM plus controls who developed an IRAE to test whether circulating cytokine levels may be predictive of developing any IRAE (Figure 5).

When compared with controls without IRAE, before ICI treatment, IRAE patients had significantly higher levels of IL-2, IL-6, IL-17A, CCL2, and free TGF-β before commencing ICI therapy (all *P* < 0.05; Figure 5, E, F, J–L, all Kruskal-Wallis with Dunn’s correction for multiple comparisons). Levels of IFNγ, TNFα, IL1β, and interleukins 4, 8, 10, and 12 did not differ between patients with and without irAE (Figure 5, A–D and G–I).

Patients who experienced IRAEs after ICI exposure had higher baseline IL-2 (Figure 5E), IL-6 (Figure 5F), IL-17A (Figure 5J), CCL2 (figure 5K) and CXCL10 (Figure 5M) levels than did those on ICI who did not experience IRAEs.

### People who develop CIADM have a more activated immune system at baseline.

**Figure 6:**
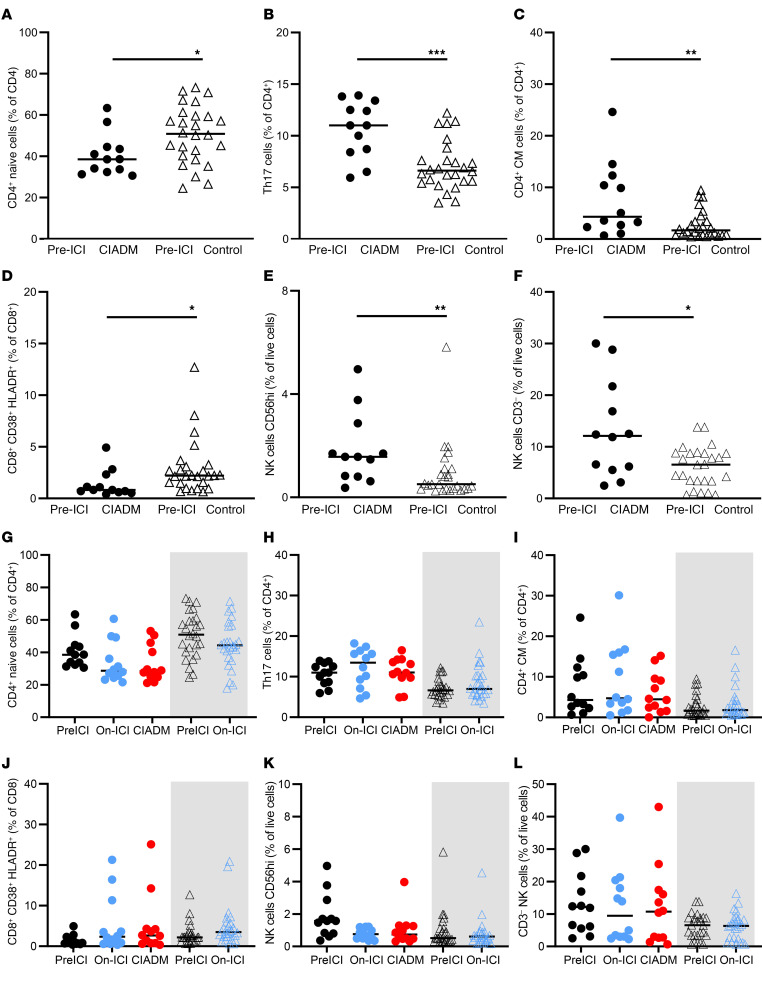
Immune cell subsets determined by flow cytometry. (**A**–**F**) Pre-ICI differences between patients with CIADM and control patients. (**G**–**L**) Data across all time points. *P < 0.05, **P < 0.01, and ***P < 0.001 for the indicated comparison, by Kruskal-Wallis test with Dunn’s correction for multiple comparisons. Lines indicate the median.

Immuno-phenotyping of circulating PBMCs revealed significant differences at baseline between patients who developed CIADM and control individuals. Patients with CIADM, before ICI exposure, had fewer naive CD4^+^ T cells (Figure 6A, *P* < 0.05), more Th17 cells (Figure 6B, *P* = 0.001), and more CD4^+^ central memory (CM) cells (*P* < 0.01, Figure 6C). Pre-ICI patients with CIADM also had fewer activated CD8^+^CD38^+^HLADR^+^ T cells (Figure 6D, *P* < 0.05).

Interestingly, given the fulminant phenotype of diabetes in many patients with CIADM, there were also differences in baseline NK cells, with more CD56^hi^ NK cells (Figure 6E, *P* < 0.01). These are an NK cell subtype more strongly associated with cytokine and chemokine production (Figure 6F, *P* < 0.05).

**Figure 7:**
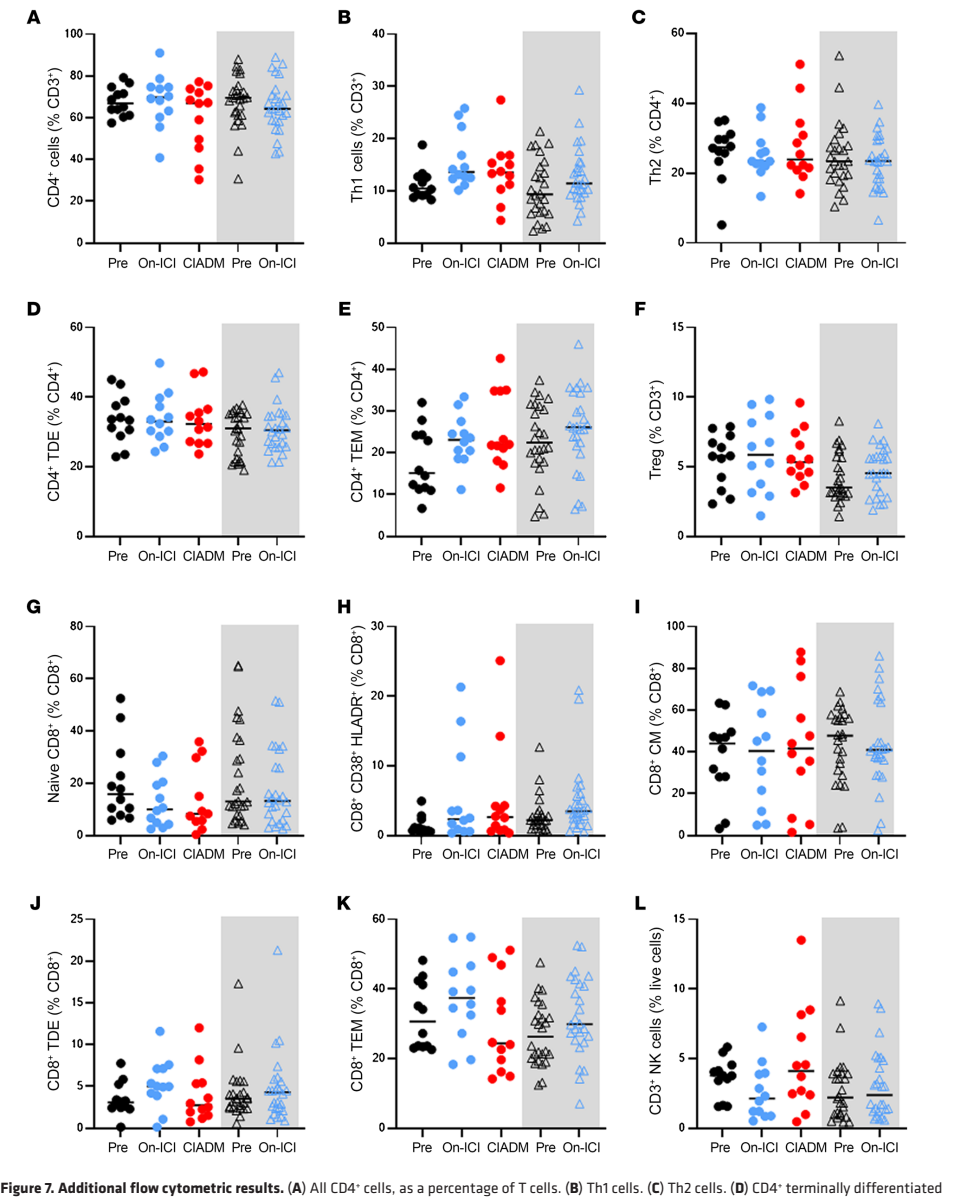
Additional flow cytometric results. (**A**) All CD4^+^ cells, as a percentage of T cells. (**B**) Th1 cells. (**C**) Th2 cells. (**D**) CD4^+^ terminally differentiated effector (TDE) cells. (**E**) CD4^+^ Tem (TEM) cells. (**F**) Tregs. (**G**) CD8^+^ naive cells. (**H**) Innate-like bystander activated T cells (CD8^+^CD38^+^HLADR^+^). (**I**) CD8^+^ CM cells. (**J**) CD8^+^ TDE cells. (**K**) CD8^+^ TEM cells. (**L**) CD3^+^ NK cells. The gray-shaded areas show data for control ICI-treated patients. No differences were statistically significant after correction for multiple comparisons.

**Figure 8:**
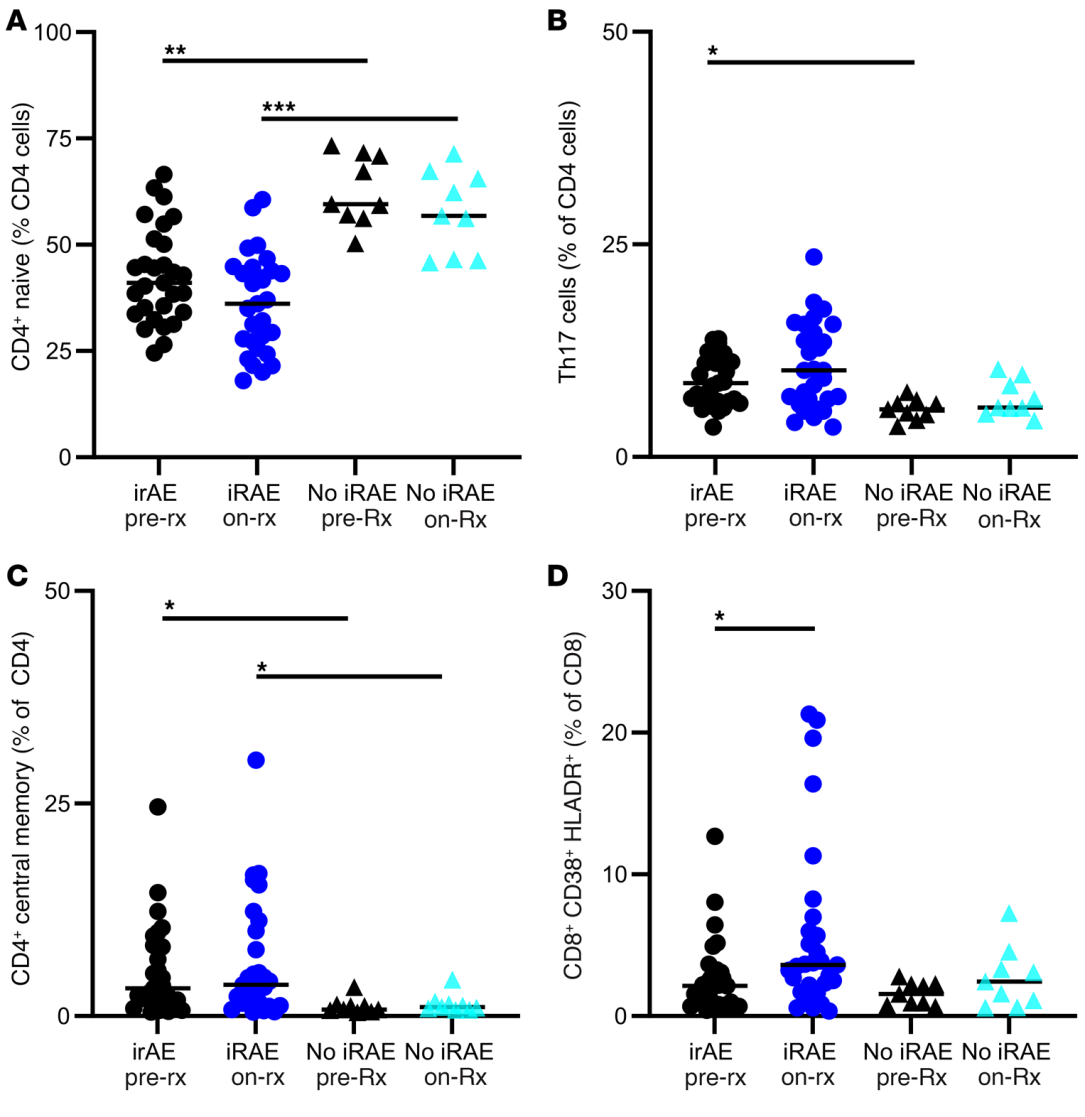
Immune cell subtypes in people with and without IRAEs. (**A**) CD4^+^ naive T cells. (**B**) Th17 cells. (**C**) CD4^+^ CM cells. (**D**) CD8^+^CD38^+^HLADR^+^ T cells. *P < 0.05, **P < 0.01, and ***P < 0.001 for the indicated comparisons, by Kruskal-Wallis test with correction for multiple comparisons.

After ICI treatment, there were no further significant changes in these cells (Figure 6, G–L). Other cell subsets including Tregs were not significantly altered (Figure 7). Figure 8 shows other flow cytometry results for cell types that were not significantly altered.

### Immune cell phenotypes also differ with IRAEs.

Flow cytometric parameters were compared between patients who experienced IRAEs and those who did not (Figure 8). At baseline, people who went on to develop an IRAE also had fewer naive CD4^+^ T cells at baseline, more Th17 cells, and more CD4^+^ CM cells (Figure 8, A–C). After ICI treatment, people with IRAEs showed an increase in CD8^+^CD38^+^HLADR^+^ cells (Figure 8D).

### Differential gene expression in CD8^+^ T cells in patients with CIADM.

CD8^+^ T cells are thought to be the major mediator of β cell death in T1D. Circulating CD8^+^ T cells were collected and RNA expression was profiled with RNA-seq. Surprisingly, before ICI therapy, there were no differentially expressed genes that passed a FDR of less than 0.05 when comparing CIADM and control patients. When we compared on-ICI controls with on-ICI CIADM patients before diagnosis, only 2 genes passed the FDR: RNF220 and BCR (both *P* = 0.044). When on-ICI controls were compared with after-diagnosis CIADM patients, no genes passed the FDR testing (Gene Expression Omnibus [GEO] accession GSE314698).

### Receiver operating characteristic curve analyses of key predictors.

**Figure 9:**
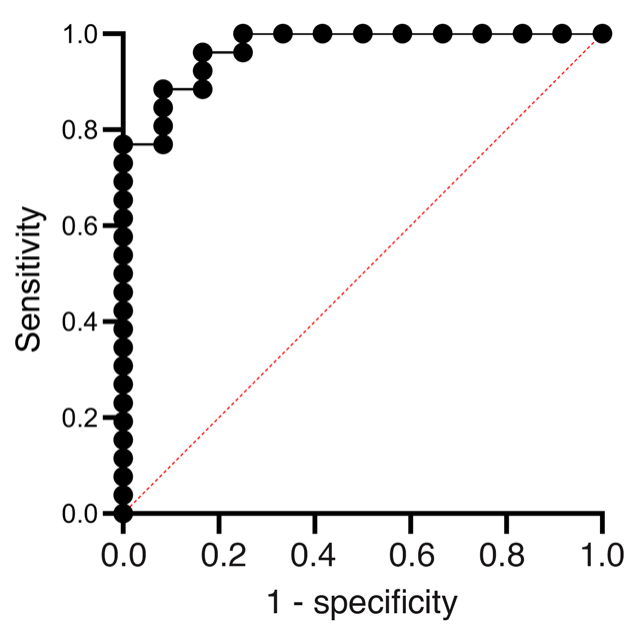
ROC curve for multiple logistic regression. An ROC curve for multiple logistic regression was generated to predict CIADM diagnosis by combining baseline anti-GAD antibody levels, anti-IAA antibody levels, pancreatic volume, CD4^+^ CM cells, CD4^+^ naive cells, Th17 cells, CD8^+^HLA-DR^+^CD38^+^ T cells, and NK CD56^hi^ cells.

The baseline variables that were significantly associated with CIADM development (anti-GAD antibody levels, anti-IAA antibody levels, pancreatic volume, CD4^+^ CM cells, CD4^+^ naive cells, Th17 cells, CD8^+^HLA-DR^+^CD38^+^ cells, and NK CD56^hi^ cells) are shown in Supplemental Table 1. They were combined in a multiple logistic regression model. Figure 9 shows that this gave a receiver operating characteristic (ROC) curve with an AUC of 0.968 (95% CI: 0.919–1.0, *P* < 0.0001). This result was associated with a positive predictive value of 92.6% and a negative predictive value of 90.91%.

The data were separately analyzed using only antibodies and pancreatic volume, as clinical flow cytometry testing may not be available in all centers in a clinically meaningful timeframe. Including only anti-GAD, anti-IAA, and pancreatic volume in the model gave an ROC curve with an AUC of 0.891 (*P* = 0.0001), with a negative predictive value of 77.8% and a positive predictive value of 82.8%.

## Discussion

Here, we report a number of baseline predictors of CIADM. Using serial samples, patients and controls were evaluated using a combination of flow cytometry, cytokine expression, autoantibody analysis, RNA-seq, and CT imaging analysis. We found that patients with CIADM had higher levels of anti-GAD and anti-IAA antibodies at baseline and lower baseline pancreatic volume compared with matched controls. Patients with CIADM had higher baseline frequencies of Th17^+^ cells and CD4^+^ CM cells and lower frequencies of naive CD4^+^ cells than did controls. Patients with CIADM also exhibited differences in lymphocyte activation-markers early on in treatment, with higher frequencies of activated CD4^+^CD38^+^HLA-DR^+^ cell subsets and lower frequencies of naive CD4^+^ cell subsets compared with controls.

In humans, limited data are available regarding the immunophenotype of CIADM. Hughes et al. reported a case series of 5 patients with CIADM, and among the 4 patients who had HLA-A2^+^ haplotyping, 2 had increased diabetes antigen–specific T cells, which were predominantly effector or memory cells (28). A mass cytometry–based study of 28 patients with melanoma treated with ICI included 2 patients with new-onset T1DM, which we would term CIADM. This study identified higher numbers of activated CD4^+^ cells in those with severe IRAEs of all types on treatment, similar to our study, but conversely to our findings, the researchers found higher numbers of naive CD4^+^ T cells to be associated with more severe IRAEs (29). However, of the 2 patients with CIADM included, we observed no significant differences compared with controls.

When looking at IRAE studies in general, Lozano et al. used single-cell RNA-Seq for T cell phenotyping in patients treated with ICIs for melanoma and found baseline and early on-treatment expansion in CD4^+^ T effector memory (Tem) cell subsets to be associated with severe IRAEs of all types, whereas in our study, CD4^+^ CM cell subsets defined by flow cytometry had the strongest association with CIADM at baseline (30). Bukhari et al. performed single-cell sequencing, which showed that PBMCs from patients with thyroiditis had higher baseline numbers of Th17 cell subsets (31). Kim et al. similarly found higher baseline numbers of Th17 subsets to be associated with the development of severe IRAEs of all types in a cohort of patients treated with ICIs for non–small cell lung cancer and thymic epithelial tumors (32).

In T1D, both Th1 and Th17 pathways are acknowledged as direct drivers of disease pathogenesis in human and animal studies (33–35). A recent study found that ustekinumab, which binds IL-12 and IL-23 to target Th1 and Th17 cells, was able to preserve pancreatic β cell function in adolescents with recent-onset T1D (36). We found increased baseline Th17 cell numbers in patients with CIADM and associated significant increases in cytokines associated with the Th17 pathway including IL-6, TGF-β, TNF-α, and IFN-δ in patients with CIADM compared with controls. Interestingly, the majority of changes in circulating immune cells were observed in CD4^+^ T cells. Consistent with this, there were essentially no changes in gene expression in circulating CD8^+^ T cells in patients with CIADM. The lack of changes in circulating CD8^+^ T cells was surprising and suggests either that CD8^+^ T cells are not important in CIADM or, more probably, that the cells of relevance were not in circulation. In T1D, the pathogenic CD8^+^ T cells are highly concentrated in the pancreas and pancreatic lymph nodes (37).

In comparison with other IRAEs, one of the unique aspects of CIADM is that its de novo counterpart, T1D, has well-established biomarkers in the form of islet autoantibodies, especially anti-GAD, anti-IA2, anti-IAA, and anti-ZnT8 autoantibodies. It is known that patients with CIADM at diagnosis have a lower prevalence of those autoantibodies than do those with T1D (4). We and others have reported pretreatment anti-GAD antibody positivity in a small proportion of patients with CIADM, but this has not been extensively tested or compared with controls (38–41). Anti-GAD antibody positivity is present in a small proportion of the general population, with a median specificity of anti-GAD antibodies in the Islet Autoantibody Accreditation Program of 98.9% (42). A Norwegian study of over 4,000 individuals found that anti-GAD antibodies have a prevalence of 1.7% in the nondiabetic Norwegian adult population (43), in which it was associated with thyroid autoimmunity. Anti-GAD antibody titers were higher in individuals with prediabetes than in those with normal metabolic parameters. Our study used the highly sensitive agglutination PCR assay to detect subclinical levels of anti-GAD antibodies, which were significantly higher than in controls and were associated with progression to CIADM. It is plausible that patients with CIADM have a subclinical degree of anti-islet autoimmunity, as evidenced by low titers of anti-GAD and anti-IAA antibodies reflecting subclinical islet autoimmunity that places these individuals at risk once exposed to ICIs.

Our population of patients with CIADM had a 66% rate of T1D risk HLA haplotypes among those who were tested. This rate is not substantially different from the background population rates of HLA-risk alleles and is substantially less than the 90%–95% rate of high-risk alleles in individuals with T1D. It is worth noting that there is variability between the frequency and composition of risk alleles in different series worldwide. This may relate, at least in part, to differences in HLA types and T1D risk alleles between people of different ethnicities.

The use of pancreatic volumetry as a biomarker for prevalent T1D and CIADM is established. Previous studies (20, 44, 45) in individuals at high risk of T1D have shown reduced pancreatic volume with progression to diabetes. Several studies have corroborated that CIADM is associated with a decline in pancreatic volume, however, baseline, pre-ICI pancreatic volumes have not previously been reported for patients compared with controls (6, 46–48).

The strengths of this work lie in the inclusion of longitudinal case control–matched samples obtained and use of a diverse range of biomarker methodologies. The biomarkers we used in our final prediction model were all noninvasive, scalable, and easily accessible clinically through peripheral blood collection and CT scans that were already being conducted as part of routine care. Automated pancreatic volumetry methodology has previously been validated (49).

The limitations of this study include the relatively low sample size, due to the relatively low incidence of CIADM (0.4%–1.9%) among anti–PD-1/anti–PD-L1 ICI–treated patients (5–9). Even so, this is the largest series of patients with CIADM with longitudinal sample analyses. The lack of significantly differentially expressed genes by RNA-seq of CD8^+^ T cells was surprising. However, most of the flow cytometry–identified differences were in CD4^+^ T cells. After the CD8^+^ T cell results were analyzed, the CD4^+^ T cells were no longer available to sequence, which is a limitation of this study. Inclusion of a T1D genetic risk score (17) may further improve the ability to predict CIADM, but this test is not routinely available.

A future validation study would allow testing of the robustness of the predictive variables identified in this study. Expanding the patient population to include other primary tumor types would be of interest. The sensitive autoantibody detection assay used in a study using an agglutination PCR assay (50) found that 64% of patients with CIADM have assay-detectable anti-GAD antibody levels versus 14% of controls (*P* < 0.001) and 54% have anti-insulin antibodies versus 21% of controls (*P* = 0.038). Use of this assay, or another similarly sensitive assay, and combining those results with CT or MRI examinations of pancreatic volume would allow for the most easily testable hypothesis. Although the circulating immune cell phenotypes add substantially to the predictive value, measurement of these would not be available at all centers.

The detection of subclinical anti-GAD antibody titers and lower baseline pancreatic volume in our CIADM cohort suggests that patients with CIADM have prior anti-islet immune responses that are poised under permissive conditions (i.e., immune checkpoint inhibition) to cause disease. That these patients had not developed T1D prior to the introduction of an anti–PD-1 or anti–PD-L1 inhibitor indicates that this immune pathway plays an important role in suppressing islet autoimmunity. The findings of higher numbers of Th17 cells and CD4^+^ CM cells and lower numbers of CD4^+^ naive T cells at baseline with more activation upon ICI introduction gives the impression of a more experienced and autoreactive immune system in patients with CIADM compared with controls without IRAEs. Combined, these findings suggest that patients with CIADM have a distinct immune profile that can be detected prior to ICI use.

The ability to predict IRAEs has unique potential when the clinical indication for ICI is not definitively superior to the alternatives. For example, in stage III melanoma, ICIs are currently considered alongside targeted therapy such as dabrafenib plus trametinib as effective adjuvant therapy, and specific contraindications to ICIs such as autoimmune disease or immunosuppressive treatment may guide choice of therapy (51). In this scenario, the ability to identify individuals at high risk of severe IRAEs could further guide therapeutic choices in this area and reduce IRAE-related morbidity. In people with a high likelihood of therapeutic benefit from ICIs but also a high risk of CIADM, knowledge of this risk would facilitate closer monitoring and, thereby, potentially prevent late diabetes diagnosis when ketoacidosis is present.

### Conclusions.

IRAEs are common in individuals treated with ICIs and vary in severity from mild to fatal. Prediction of IRAEs prior to therapy has the potential to inform clinical decisions, allow for earlier detection, and open a potential window for prevention. By combining biomarkers from the fields of T1D and IRAE research, we have identified biomarkers that have the potential to predict checkpoint inhibitor–related autoimmune diabetes from baseline and on-treatment characteristics. Prospective validation of these biomarkers is a crucial next step but a challenging prospect, given the relatively low incidence of CIADM.

## Methods

### Sex as a biological variable.

Of the patients studied, 11 of these individuals with CIADM were male and 3 were female, and 22 of the ICI-treated control participants were male and 6 were female. Melanoma has a higher incidence in male individuals.

### Sample selection.

Fourteen patients with CIADM and 28 ICI-treated controls who had longitudinal biospecimens were identified from the prospectively collected Melanoma Institute of Australia medical record database (MRD2) and biospecimen bank.

The diagnosis of CIADM was based on new-onset diabetes (hemoglobin A1c [HbA1c] ≥6.5% and/or blood glucose ≥11 mmol/L) in the setting of ICI therapy, with evidence of insulin deficiency (either the presence of diabetic ketoacidosis or low C-peptide levels of ≤0.4 nmol/L with elevated glucose). No patients had previous diabetes diagnosis.

Two controls were selected for each patient with CIADM, matched as closely as possibly for age (±5 years), sex, type of ICI therapy (single-agent anti–PD-1 vs. combined anti–cytotoxic T lymphocyte–associated protein 4 [anti-CTLA4] plus anti–PD-1), duration of therapy, treatment response, and concurrent other IRAEs. If patients with CIADM had no other IRAEs, they were matched with controls without IRAEs. If patients with CIADM had other IRAEs, they were matched with controls who had those same IRAEs, or if no such controls could be found, then with a control who had no IRAEs.

Analysis was done of control patients’ prospectively collected pre-ICI and on-ICI PBMCs (~3 months after treatment initiation) and serum samples. Patients with CIADM similarly had pre-ICI and on-treatment blood samples collected at approximately 3 months. Patients with CIADM additionally had samples taken approximately 3 months after CIADM diagnosis. A subgroup of control patients did not develop any IRAEs, and they were also separately compared to assess the effect of general ICI-related immune changes for various parameters. A summary of the methods is depicted in Figure 1.

### Autoantibody analysis.

T1D autoantibody levels (anti-GAD, anti-IA2, anti-ZnT8, and anti-IA2) in serum samples were determined using an agglutination PCR assay, which has been previously described (50). Clinical thresholds for each autoantibody are set at the 98th percentile of results from 60–84 negative serum samples included in the 2023 International Islet Autoantibody Standardization Program (52).

### Cytokine expression.

Serum cytokine expression was measured using the BioLegend LEGENDplex Human Essential Immune Response Multiplex Assay (catalog 740930). This measures the interleukins IL-1β, IL-2, IL-4, IL-6, IL-10, IL-12p70, IL-17A, IFN-γ, TNF-α, CCL2, CXCL8 (IL-8), CXCL10, and free TGF-β1. The assay was conducted in accordance with the manufacturer’s instructions, with samples run in duplicate.

### C-peptide assay.

Serum C-peptide levels were measured using the human C-peptide ELISA assay (CrystalChem, catalog 80954) according to the manufacturer’s instructions.

### Glucose levels.

Serum glucose levels were measured directly from serum samples using Abbott Freestyle Libre glucometer and glucose test strips.

### CT pancreatic volumetry.

CT pancreatic volumetry was conducted by 1 investigator, as previously published (53), using Vitrea software (Figure 1B). CT scans were obtained within 6 months of each blood collection time point. CT scans of patients with CIADM were compared with the control cohort. Some of these pancreatic volumetry results have previously been published (53), and the expanded cohort is presented. CT scans were not available for 1 patient with CIADM and 2 controls. Most patients with CIADM did not have protocol CT scans on ICI before CIADM, so data are not available for that time point.

### Flow cytometry.

Cryopreserved PBMCs were thawed in media and washed in FACS buffer prior to staining. Samples were stained first with FVS700 Viability Dye (catalog 564997) in the dark for 10 minutes, followed by human AB serum for 10 minutes. All antibodies were purchased from BD Biosciences except CXCR5 PE-Cy7, which was from BioLegend. Cells were then stained with CCR6 BV480 (catalog 556130), CXCR3 PE-CF596 (catalog 562451), CCR7 BB700 (catalog 566438), and CXCR5 PE-Cy7 (catalog 356923) at 37°C for 15 minutes. Surface staining was then performed with CD45RA APC-H7 (catalog 560674), CD8 BUV496 (catalog 612943), CD127 BV786 (catalog 563324), CD3 BUV661 (catalog 612965), CD25 BB515 (catalog 564467), CD56 BUV737 (catalog 612767), CD16 BUV563 (catalog 741449), CD4 BUV805 (catalog 612887), CD38 BV421 (catalog 562445), and HLA-DR BUV395 (catalog 565972) at 4°C for 30 minutes. Cells were analyzed using the BD Symphony Analyzer with the gating strategy as shown in Supplemental Figure 1. A minimum of 30,000 cells were analyzed per sample.

### Cell sorting.

Cryopreserved PBMCs were thawed and washed in FACS buffer prior to staining with FC block, CD45^+^, CD3^+^ CD8^+^, CD4^+^, and DAPI. CD8^+^ T cells were identified via gating for CD45^+^CD3^+^CD8^+^CD4^–^, and DAPI^–^ subsets via the BD Influx cell sorter. CD8^+^ T cells (1,000 per sample) were sorted into a 96-well plate and frozen down according to the manufacturer’s instructions.

### RNA extraction and sequencing.

Total RNA was extracted using the Ultra Low Input Takarabio kit. RNA was extracted and sequenced using a NovaSeq X with approximately 10 million 150 bp paired-end reads.

RNA-seq analysis was performed using R, edgeR was used for differential gene analysis, and STAR, RSEM, Tximport, and DESeq2 with a Gencode 45 (latest) annotation were used for isoform analysis. Flow cytometric data were analyzed using FlowJo.

### Statistics.

Statistical analysis was performed using SPSS version 21 (IBM) or GraphPad Prism 10 (GraphPad Software). Most serum, cytokine and flow cytometric data were not normally distributed and were compared with a Mann-Whitney *U* test where only 2 datasets were compared, or by Kruskal-Wallis test with Dunn’s correction for multiple comparisons where more than 2 sets were examined. In the case of matching samples, e.g., pre-ICI, on-ICI, and CIADM in the same individuals, a Wilcoxon signed-rank test was used, again, corrected for multiple comparisons. Normally distributed data were compared using 1-way ANOVA with correction for multiple comparisons. Multiple-comparison–adjusted *P* values of less than 0.05 were considered statistically significant. When comparing paired data across time courses, the Wilcoxon signed-rank test was used with manual addition of the correction for the number of comparisons with Bonferroni. SPSS Statistics version 28 was used to analyze the variables demonstrating univariate association with diabetes status. These were candidates for inclusion in multivariate binary logistic regression models. Backward stepwise variable selection was used to identify the independent predictors of diabetes status in the best-fitting multivariate logistic regression model. The area under the ROC was utilized to evaluate the performance of the fitted model from the best multivariate logistic regression model to correctly classify a patient’s diabetes status. *P* values of less than 0.05 after any corrections for multiple comparisons were considered significant. Illustrations were made using Adobe Illustrator or GraphPad Prism. Data are presented as individual points (all Figures) with medians (Figure 2 and Figures 4–8).

### Study approval.

All patients gave written informed consent. The study was approved by the Royal Prince Alfred Hospital Research Ethics Committee, Sydney (protocol no. X10-0305 and HREC/10/RPAH).

### Data availability.

Data other than the original sequencing files are available in the NCBI’s GEO database (GSE314698) and via FigShare using doi 10.6084/m9.figshare.29453093. Normalized RNA-seq data are available at the same FigShare location. The original sequencing files can be obtained from the corresponding author. Values for all data points in graphs are reported in the Supporting Data Values file.

## Author contributions

LW, JMW, CL, NF, MC, DAB, RCB, GVL, RAS, SCS, VHMT, AMM, and JEG contributed to the study’s design. Patients were recruited to the database and biobank by MC, GVL, RAS, and AMM, LW, JMW, and NF conducted experiments and acquired data. Data analysis was performed by LW, JMW, CL, NF, NN, and JEG. Figures were prepared by LW, NN, and JEG. All authors contributed to the writing and revision of the manuscript.

## Funding support

- National Health and Medical Research Council (NHMRC) Investigator grant 2033228 (to JEG).
- NHMRC Program grant 1149976 (to JEG).
- NHMRC Investigator grant 2009476 (to AMM).
- NHMRC Investigator grant 2007839 (to GVL).

## Supplementary Material

Supplemental data

ICMJE disclosure forms

Supporting data values

## Figures and Tables

**Table 1:**
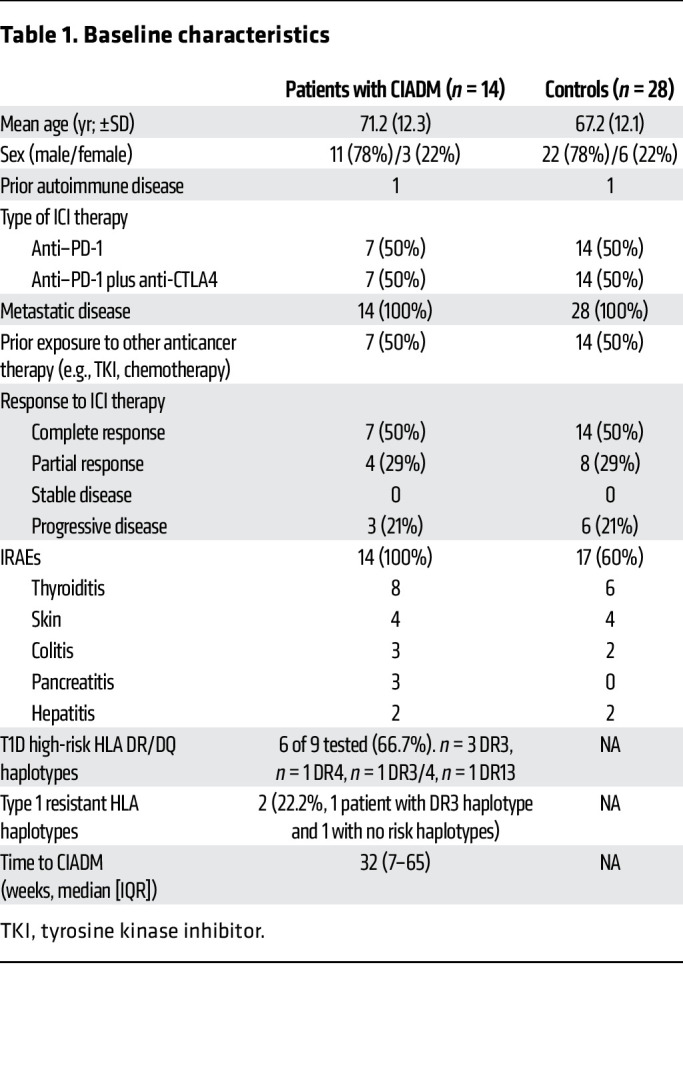
Baseline characteristics

## Version 1 11/20/2025

In-Press Preview

## Version 2 02/16/2026

Electronic publication

## References

[1] Paul J (2024). Overlapping and non-overlapping indications for checkpoint inhibitors in the US. J Clin Oncol.

[2] Menzies AM (2019). Pathological response and survival with neoadjuvant therapy in melanoma: a pooled analysis from the international neoadjuvant melanoma consortium (INMC). Nat Med.

[3] Patel SP (2023). Neoadjuvant-adjuvant or adjuvant-only pembrolizumab in advanced melanoma. N Engl J Med.

[4] Wu L (2023). Risk factors and characteristics of checkpoint inhibitor-associated autoimmune diabetes mellitus (CIADM): a systematic review and delineation from type 1 diabetes. Diabetes Care.

[5] Tsang VHM (2019). Checkpoint inhibitor-associated autoimmune diabetes is distinct from type 1 diabetes. J Clin Endocrinol Metab.

[6] Byun DJ (2020). Immune checkpoint inhibitor-associated diabetes: a single-institution experience. Diabetes Care.

[7] Wright JJ (2018). Increased reporting of immune checkpoint inhibitor-associated diabetes. Diabetes Care.

[8] De Filette JMK (2019). Immune checkpoint inhibitors and type 1 diabetes mellitus: a case report and systematic review. Eur J Endocrinol.

[9] Barroso-Sousa R (2018). Incidence of endocrine dysfunction following the use of different immune checkpoint inhibitor regimens: a systematic review and meta-analysis. JAMA Oncol.

[10] Chaput N (2017). Baseline gut microbiota predicts clinical response and colitis in metastatic melanoma patients treated with ipilimumab. Ann Oncol.

[11] Nuñez NG (2023). Immune signatures predict development of autoimmune toxicity in patients with cancer treated with immune checkpoint inhibitors. Med.

[12] Subudhi SK (2016). Clonal expansion of CD8 T cells in the systemic circulation precedes development of ipilimumab-induced toxicities. Proc Natl Acad Sci U S A.

[13] Oh DY (2017). Immune toxicities elicted by CTLA-4 blockade in cancer patients are associated with early diversification of the T-cell repertoire. Cancer Res.

[14] Khan S (2019). Immune dysregulation in cancer patients developing immune-related adverse events. Br J Cancer.

[15] Tarhini AA (2015). Baseline circulating IL-17 predicts toxicity while TGF-β1 and IL-10 are prognostic of relapse in ipilimumab neoadjuvant therapy of melanoma. J Immunother Cancer.

[16] Fujisawa Y (2017). Fluctuations in routine blood count might signal severe immune-related adverse events in melanoma patients treated with nivolumab. J Dermatol Sci.

[17] Ruiz-Esteves KN (2024). Identification of immune checkpoint inhibitor–induced diabetes. JAMA Oncol.

[18] Ziegler AG (2013). Seroconversion to multiple islet autoantibodies and risk of progression to diabetes in children. JAMA.

[19] Redondo MJ (2018). A type 1 diabetes genetic risk score predicts progression of islet autoimmunity and development of type 1 diabetes in individuals at risk. Diabetes Care.

[20] Virostko J (2024). Longitudinal assessment of pancreas volume by MRI predicts progression to stage 3 type 1 diabetes. Diabetes Care.

[21] Shapiro MR (2023). Human immune phenotyping reveals accelerated aging in type 1 diabetes. JCI Insight.

[22] Kenefeck R (2015). Follicular helper T cell signature in type 1 diabetes. J Clin Invest.

[23] Ferreira RC (2015). IL-21 production by CD4^+^ effector T cells and frequency of circulating follicular helper T cells are increased in type 1 diabetes patients. Diabetologia.

[24] Shek D (2020). Non-coding RNA and immune-checkpoint inhibitors: friends or foes?. Immunotherapy.

[25] Long SA (2010). Defects in IL-2R signaling contribute to diminished maintenance of FOXP3 expression in CD4(^+^)CD25(^+^) regulatory T-cells of type 1 diabetic subjects. Diabetes.

[26] Mallone R (2007). CD8^+^ T-cell responses identify beta-cell autoimmunity in human type 1 diabetes. Diabetes.

[27] Herold KC (2009). Validity and reproducibility of measurement of islet autoreactivity by T-cell assays in subjects with early type 1 diabetes. Diabetes.

[28] Hughes J (2015). Precipitation of autoimmune diabetes with anti-PD-1 immunotherapy. Diabetes Care.

[29] Kovacsovics-Bankowski M (2024). Lower frequencies of circulating suppressive regulatory T cells and higher frequencies of CD4^+^ naive T cells at baseline are associated with severe immune-related adverse events in immune checkpoint inhibitor-treated melanoma. J Immunother Cancer.

[30] Lozano AX (2022). T cell characteristics associated with toxicity to immune checkpoint blockade in patients with melanoma. Nat Med.

[31] Bukhari S (2023). Single-cell RNA sequencing reveals distinct T cell populations in immune-related adverse events of checkpoint inhibitors. Cell Rep Med.

[32] Kim KH (2020). Immune-related adverse events are clustered into distinct subtypes by T-cell profiling before and early after anti-PD-1 treatment. Oncoimmunology.

[33] Honkanen J (2010). IL-17 immunity in human type 1 diabetes. J Immunol.

[34] Kuriya G (2013). Double deficiency in IL-17 and IFN-γ signalling significantly suppresses the development of diabetes in the NOD mouse. Diabetologia.

[35] Reinert-Hartwall L (2015). Th1/Th17 plasticity is a marker of advanced β cell autoimmunity and impaired glucose tolerance in humans. J Immunol.

[36] Tatovic D (2024). Ustekinumab for type 1 diabetes in adolescents: a multicenter, double-blind, randomized phase 2 trial. Nat Med.

[37] Kent SC (2005). Expanded T cells from pancreatic lymph nodes of type 1 diabetic subjects recognize an insulin epitope. Nature.

[38] Gauci ML (2017). Autoimmune diabetes induced by PD-1 inhibitor-retrospective analysis and pathogenesis: a case report and literature review. Cancer Immunol Immunother.

[39] Godwin JL (2017). Nivolumab-induced autoimmune diabetes mellitus presenting as diabetic ketoacidosis in a patient with metastatic lung cancer. J Immunother Cancer.

[40] Lowe JR (2016). Genetic risk analysis of a patient with fulminant autoimmune type 1 diabetes mellitus secondary to combination ipilimumab and nivolumab immunotherapy. J Immunother Cancer.

[41] Stamatouli AM (2018). Collateral damage: insulin-dependent diabetes induced with checkpoint inhibitors. Diabetes.

[42] Lampasona V (2019). Islet autoantibody standardization program 2018 workshop: interlaboratory comparison of glutamic acid decarboxylase autoantibody assay performance. Clin Chem.

[43] Sørgjerd EP (2015). Presence of anti-GAD in a non-diabetic population of adults; time dynamics and clinical influence: results from the HUNT study. BMJ Open Diabetes Res Care.

[44] Williams AJK (2012). Pancreatic volume is reduced in adult patients with recently diagnosed type 1 diabetes. J Clin Endocrinol Metab.

[45] Roger R (2022). Deep learning-based pancreas volume assessment in individuals with type 1 diabetes. BMC Med Imaging.

[46] Marchand L (2019). Diabetes mellitus induced by PD-1 and PD-L1 inhibitors: description of pancreatic endocrine and exocrine phenotype. Acta Diabetol.

[47] Wei HH (2024). Distinct changes to pancreatic volume rather than pancreatic autoantibody positivity: insights into immune checkpoint inhibitors induced diabetes mellitus. Diabetol Metab Syndr.

[48] Perdigoto AL (2022). Immune cells and their inflammatory mediators modify β cells and cause checkpoint inhibitor-induced diabetes. JCI Insight.

[49] Lim SH (2022). Automated pancreas segmentation and volumetry using deep neural network on computed tomography. Sci Rep.

[50] Sing ABE (2024). Feasibility and validity of in-home self-collected capillary blood spot screening for type 1 diabetes risk. Diabetes Technol Ther.

[51] Funck-Brentano E (2021). Which adjuvant treatment for patients with BRAF^V600^-mutant cutaneous melanoma?. Ann Dermatol Venereol.

[52] Marzinotto I (2023). Islet Autoantibody Standardization Program: interlaboratory comparison of insulin autoantibody assay performance in 2018 and 2020 workshops. Diabetologia.

[53] Wu L (2024). Checkpoint inhibitor-associated autoimmune diabetes mellitus is characterized by c-peptide loss and pancreatic atrophy. J Clin Endocrinol Metab.

