# Supplementary material for: Pancreatic volume and immune biomarkers predict checkpoint inhibitor–associated autoimmune diabetes in humans
Source: J Clin Invest. 2025 Nov 20;136(4):e192938. doi: 10.1172/JCI192938 (PMC12904724; doi:10.1172/JCI192938)
Supplement: Supplemental data [file jci-136-192938-s358.pdf]

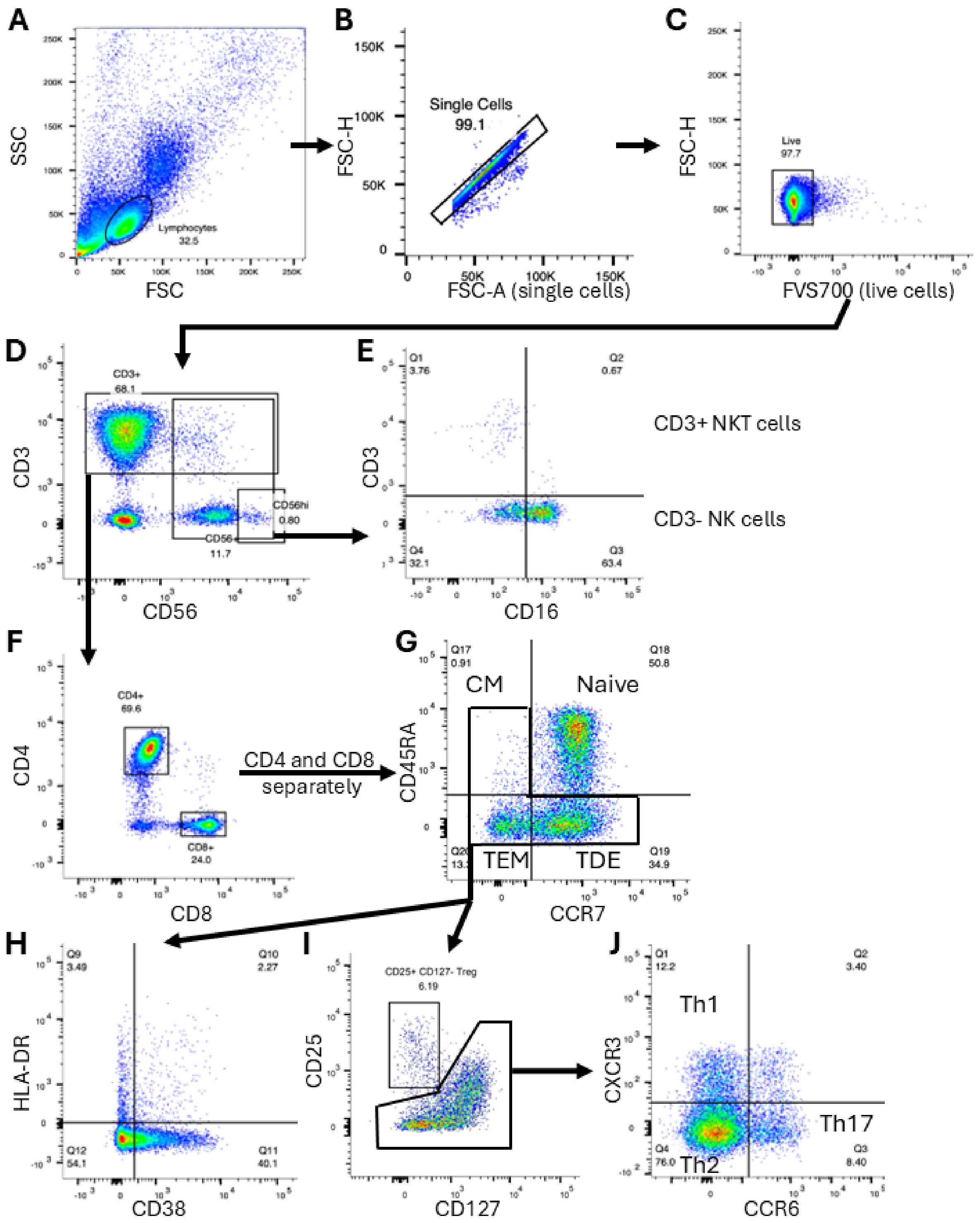

Supplementary Figure 1. Gating strategy for T and NKT phenotyping in PBMC. Cells were gated on **A)** Lymphocytes, **B)** single cells, **C)** Live cells, **D)** CD56+ cells were distinguished from CD3+ cells. **E)** CD56+ cells were gated on CD3 and CD16+ status to identify CD3+ NKT and CD3- NK cells. **F)** CD3+ cells were gated by CD4+ or CD8+. **G)** CD4+ and CD8+ T-cells were separately gated on CD45RA versus CCR7 to identify central memory (CM), naïve T cells, T effector memory (TEM) and terminally differentiated effector (TDE) cells. **H)** Activation markers HLA-DR and CD38 were used to gate % activated T cells in the CD4+ and CD8+ subsets. **I)** CD4+Cd25+ CD127- T regulatory cells were gated. **J)** CD4+ cells were gated with CXCR3 and CCR6 to identify Th1, Th2 and Th17 status.

Supplementary Table 1. Significant variables for pre-ICI comparisons. These variables were input into multiple logistic regression.

	CIADM	Control	Test, p-value
**Baseline pancreatic volume (mls)**	56±8	77±5	T-test p=0.023
**Anti-GAD**	2.9 (0-6.1)	0 (0-0.8)	M-W p=0.0021
**Anti-IAA**	2.0 (1.4-2.4)	1.0 (0-1.78)	M-W p=0.048
**% CD4+ central memory**	4.3 (2.4-11.8)	1.7 (0.7-3.7)	M-W p=0.01
**% CD4+ naïve**	38.5 (32.7-44.2)	50.8 (39.9-60)	M-W p=0.03
**% Th17 cells**	11 (8.5-13.2)	6.6 (5.6-7.9)	M-W p=0.001
**% CD8+HLA-DR+CD38+**	0.8 (0.6-2.0)	2 (1.3-3.1)	M-W p=0.014
**% NK CD56hi**	1.6 (0.8-2.6)	0.5 (0.3-1.1)	M-W p=0.0065

Data which is normally distributed is presented as mean±SEM and non-parametric data is shown with median and interquartile range (IQR). M-W = Mann Whitney
